# An Updated Insight into the Sialotranscriptome of *Triatoma infestans*: Developmental Stage and Geographic Variations

**DOI:** 10.1371/journal.pntd.0003372

**Published:** 2014-12-04

**Authors:** Alexandra Schwarz, Nora Medrano-Mercado, Günter A. Schaub, Claudio J. Struchiner, M. Dolores Bargues, Michael Z. Levy, José M. C. Ribeiro

**Affiliations:** 1 Institute of Parasitology, Biology Centre of the Academy of Sciences of Czech Republic, Ceske Budejovice, Czech Republic; 2 Laboratory of Chagas Disease and Immunoparasitology, Universidad Mayor de San Simón, Cochabamba, Bolivia; 3 Zoology/Parasitology Group, Ruhr-University Bochum, Bochum, Germany; 4 Escola Nacional de Saúde Pública. Fiocruz, Rio de Janeiro, Brazil; 5 Departamento de Parasitología, Facultad de Farmacia, Universidad de Valencia, Valencia, Spain; 6 Universidad Peruana Cayetano Heredia, Sede de Arequipa, Arequipa, Peru; 7 Department of Biostatistics and Epidemiology, Center for Clinical Epidemiology and Biostatistics, School of Medicine, University of Pennsylvania, Philadelphia, Pennsylvania, United States of America; 8 Laboratory of Malaria and Vector Research, National Institute of Allergy and Infectious Diseases, National Institutes of Health, Rockville, Maryland, United States of America; Liverpool School of Tropical Medicine, United Kingdom

## Abstract

**Background:**

*Triatoma infestans* is the main vector of Chagas disease in South America. As in all hematophagous arthropods, its saliva contains a complex cocktail that assists blood feeding by preventing platelet aggregation and blood clotting and promoting vasodilation. These salivary components can be immunologically recognized by their vector's hosts and targeted with antibodies that might disrupt blood feeding. These antibodies can be used to detect vector exposure using immunoassays. Antibodies may also contribute to the fast evolution of the salivary cocktail.

**Methodology:**

Salivary gland cDNA libraries from nymphal and adult *T. infestans* of breeding colonies originating from different locations (Argentina, Chile, Peru and Bolivia), and cDNA libraries originating from F1 populations of Bolivia, were sequenced using Illumina technology. Coding sequences (CDS) were extracted from the assembled reads, the numbers of reads mapped to these CDS, sequences were functionally annotated and polymorphisms determined.

**Main findings/Significance:**

Over five thousand CDS, mostly full length or near full length, were publicly deposited on GenBank. Transcripts that were over 10-fold overexpressed from different geographical regions, or from different developmental stages were identified. Polymorphisms were mapped to derived coding sequences, and found to vary between developmental instars and geographic origin of the biological material. This expanded sialome database from *T. infestans* should be of assistance in future proteomic work attempting to identify salivary proteins that might be used as epidemiological markers of vector exposure, or proteins of pharmacological interest.

## Author Summary

*Triatoma infestans* is the main vector of Chagas disease in South America. As in all hematophagous arthropods, its saliva contains a complex cocktail that assists blood feeding by preventing platelet aggregation and blood clotting and promoting vasodilation. These salivary components can be immunologically recognized by their hosts and targeted with antibodies that might disrupt blood feeding. The respective antibodies can be used to detect vector exposure using immunoassays. On the other hand, antibodies may also contribute to the fast evolution of the salivary cocktail. In this work, we attempted to identify variations in the salivary proteins of *T. infestans* using Illumina technology that allowed identification of over five thousand proteins based on over 300 million sequences obtained from ten salivary gland libraries. This expanded sialome database from *T. infestans* should be of assistance in future work attempting to identify salivary proteins that might be used as epidemiological markers of vector exposure, or proteins of pharmacological interest.

## Introduction

Chagas disease is endemic to Latin America [1], [2] and is caused by the protozoan parasite *Trypanosoma cruzi*, which is transmitted to humans by triatomine vectors [3]. Although there are 140 extant species of triatomine bugs, a relatively small number are implicated as human vectors, related to their adaptation to colonize human dwellings. Among these limited numbers of species, *Triatoma infestans* is recognized as an important vector in South America, being responsible for half of the disease transmission to humans. It historically covered a large geographical range, including Argentina, Chile, Brazil, Paraguay, Bolivia and Peru [4].

When attempting to feed, blood sucking animals inject saliva into their vertebrate hosts' skin to counteract their hemostasis and inflammatory reactions that might otherwise stop blood flow. In particular, anti-platelet and anti-clotting inhibitors, vasodilators and anesthetics are known to occur in these animals saliva as well as in *T. infestans* [5], [6]. Probably because of their hosts' immune pressure against salivary proteins, genes coding for salivary polypeptides in blood sucking arthropods are at a very fast pace of evolution, demonstrated for a set of salivary coding genes from the mosquito, *Anopheles gambiae*, which showed indications of positive selection [7]. Related to the fast pace of salivary protein evolution, host immune response to vectors can be quite specific and serve as an epidemiological marker of vector exposure [8]–[14].

This has also been considered for a *T. infestans* salivary antigen that might serve as an epidemiological marker of chicken exposure to this insect [15], [16]. Its recombinant form, r*Ti*SP14.6, was very effective in detecting differences in infestation levels of *T. infestans* in Bolivian households by analyzing IgG levels against the corresponding salivary protein using chicken sera [17]. IgM antibodies of chicken sera also reacted with r*Ti*SP14.6, but compared to IgG immune responses of chickens, no differences were detectable in the overall antibody reactions to either crude saliva or r*Ti*SP14.6 from sera originating from animals at low or high *T. infestans* infested households [15]. The saliva composition of hematophagous arthropods does not only differ between populations of the same species as analyzed for sand flies [18] and triatomines [19], [20], but also between developmental stages [21], [22]. Furthermore, the immune response of *T. infestans*-exposed guinea pigs varies according to the developmental stage (nymphs or adults) and the geographical origin of the colonies [23].

In order to develop an appropriate *T. infestans* exposure marker, in particular a salivary antigen that will be recognized by sera of triatomine host species exposed to any developmental stage or strain of *T. infestans*, we aim in this study to use an RNAseq strategy to determine developmental stage and geographical variations in the sialome (from the Greek sialo = saliva) of *T. infestans* that could eventually be used to design specific immunological markers of vector exposure. Additionally we aim to extend the sialome database of *T. infestans* that could be used for further functional determination of the identified salivary proteins.

## Materials and Methods

### Ethics statement

All experimental exposures of animals to triatomines carried out in the Czech Republic were in accordance with the Animal Protection Law of the Czech Republic (§17, Act No. 246/1992 Sb) and with the approval of the Academy of Science of the Czech Republic (protocol approval no. 172/2010) which complies with the regulations of the European Directive 2010/63/EU on the protection of animals used for scientific purposes in Europe.

### *Triatoma infestans* and haplotyping

All *T. infestans* strains (n = 22) originating from Argentina, Bolivia, Chile and Peru were reared in the insectary at an air temperature of 28±1°C, a relative humidity of 60–70% and with a 12 h/12 h light/dark cycle. Supplemental Table S1 summarizes the origin and the collection date of the different *T. infestans* strains from the natural settings in South America. For colony maintenance triatomines were regularly fed on guinea pigs or rabbits.

Complete sequences of ITS-1, 5.8S and ITS-2 comprising the entire rDNA intergenic region of the different *T. infestans* strains were analysed and for each *T. infestans* strain an adult and either a 4^th^ or 5^th^ nymphal stage were examined. One or two legs of each bug were used for DNA extraction using methods as previously described [24]–[26] and the primers designed for the flanking regions of the intergenic region derived from [27] and [4]. Amplification procedures and thermal cycler conditions were carried out in a Mastercycle ep Gradient (Eppendorf, Germany) using 30 cycles of 30 s at 94°C, 30 s at 50–55°C and 1 min at 72°C following 30 s at 94°C and 7 min at 72°C. Primers and nucleotides were removed from PCR products by purification using the Ultra Clean PCR Clean-up DNA Purification System (MoBio, USA) according to the manufacturer's protocol. Amplified DNA was resuspended in 50 µl of 10 mM TE buffer (pH 7.6) and the final DNA concentration was determined by measuring the absorbance at 260 nm and 280 nm. Sequencing of the complete intergenic region was performed on both DNA strands by the dideoxy chain-termination method using the Taq dye-terminator chemistry kit for ABI 3130 and ABI 3700 (Applied Biosystems, USA) and PCR primers. Given the importance of the recent discovery of a pseudogenic 5.8S+ITS-2 sequence, named as “ps(5.8S+ITS-2)”, that is widely distributed in triatomines of North, Central and South America [28], it was assured that no double sequencing signal was present in the chromatograms in order to confirm that variable positions in the intergenic region were not due to an underlying paralogous sequence.

The haplotype (H) terminology used for both ITS haplotypes followed the nomenclature for composite haplotyping (CH) as previously proposed [4], [29], [30]. Accordingly, ITS-2 haplotypes are labelled by numbers and ITS-1 haplotypes by capital letters. Sequences were aligned with CLUSTAL W2 [31] and molecular evolutionary analyses were conducted using MEGA, version 6 [32] and default parameters, including gap penalties in pairwise and multiple alignments. NCBI BLAST (Basic Local Alignment Search Tool, http://blast.ncbi.nlm.nih.gov/Blast.cgi) was used for sequence comparison against the GenBank database. Length and GC-content of each marker sequence including the complete intergenic rDNA region were determined by PAUP 4.0b10 [33] and MEGA 6 programs. Triatominae sequence comparisons were performed using complete or almost complete sequences of the same molecular markers available in GenBank-EMBL. The following sequences have been used: A) rDNA ITS1-5.8S-ITS2, complete region: *T. infestans* GT1A, GT2A, GT3A, GT4A, GT5A, GT1B, GT1C (AJ576051- AJ576055; AJ582024, AJ582025); *Triatoma platensis* GT1A, GT1B (AJ576061, AJ576062); *Triatoma delpontei* GT1A, GT2A, GT3A, GT3B (AJ576057- AJ576060); *Triatoma melanosoma* GT1A (AJ576056) [4]; B) rDNA ITS-1, complete or almost complete region: *T. infestans* haplotypes D and E (HQ437705, HQ437706) [34]; *T. infestans* clone TiITS10037 (DQ118960) (unpublished); C) rDNA ITS-2, complete or almost complete region: *T. infestans* Hap1, Hap2, Hap3, Hap4 (HQ333211–HQ333214) [35]; *T. infestans* isolate ITInf72 and ITInf74 (AY860387, AY860388) [36].

### Triatomine exposure experiments, salivary gland dissections and total RNA isolations

All exposure experiments were performed with starved triatomines of 14 out of 22 *T. infestans* strains. These triatomines had starved for two weeks following moulting. Guinea pigs were obtained from our in-house breeding of the animal facility for the triatomine feeding experiments and rabbits from Velaz, Únětice, Czech Republic. As summarized in Supplemental Table S1 seven out of the 14 *T. infestans* strains were fed on guinea pigs and the other 7 strains were fed on rabbits. A total of 1876 triatomines were fed once until a full blood meal was completed (Supplemental Table S2); feeding times depended on the developmental stage and were about 45–60 min using guinea pigs and 15–30 min using rabbits.

Triatomines were dissected in sterile DEPC/PBS buffer and the salivary glands (SG) transferred into lysis buffer RA1 of the NucleoSpin RNA and RNA XS kits (Macherey-Nagel, Germany). The salivary glands of 2 individuals of each developmental stage and strain of *T. infestans* were dissected after 6 h, 12 h, and 24 h after feeding followed by additional dissections every 2 days after the triatomine blood meal until each developmental stage moulted into the next stage (see Supplemental Table S2 for development times). Adults (1 female and 1 male per dissection time point) were dissected until female *T. infestans* started to lay eggs (about 12 days after a blood meal) and SGs of starved *T. infestans* of all instars and adults were additionally prepared. A total of 1,624 nymphs and 252 adults were dissected and the glands were stored at −70°C. No normalization of the RNA was made, since the pools had equal representation of tissues from their instar developmental time.

From these insects, a total of 10 pooled total RNA samples were prepared for cDNA library constructions and next generation sequencing following the NucleoSpin RNA and RNA XS kits (Macherey-Nagel) manufacturer's instructions (Supplemental Table S1 and Table 1). In particular, 4 total RNA samples contained SG RNA of adults from colony *T. infestans* strains of Chile (Chile-A, 18 bugs dissected), Peru (Peru-A, 36 bugs dissected), Argentina (Arg-A,18 bugs dissected) and Bolivia (BolCol-A, 108 bugs dissected) and 4 other RNA samples were prepared from nymphal SG RNA of also colony strains from Chile (Chile-N, 116 bugs dissected), Peru (Peru-N, 232 bugs dissected), Argentina (Arg-N, 116 bugs dissected) and Bolivia (BolCol-N, 696 bugs dissected). The further 2 RNA samples were set up of either adult SG RNA from the Bolivian *T. infestans* strain collected in 2012 (BolNat-A, 72 bugs dissected) or of nymphal SG RNA from field collected *T. infestans* (BolNat-N, 464 bugs dissected). Each RNA sample contained total RNA from all SGs of all developmental stages of the different *T. infestans* strains dissected 6 h after a blood meal until the triatomines moulted (covering the entire life cycle) and/or started to lay eggs as in the case of female *T. infestans*. The total RNA concentration of all 10 samples was measured using the NanoDrop spectrophotometer (Thermo Fisher Scientific, USA), and the RNA of each sample was precipitated with ethanol using a final concentration of 0.3 M Na-acetate, pH 5.2. Precipitated RNA was overlaid with 80% ethanol and stored at −70°C.

**Table 1:** RNAseq metadata from *T. infestans* sialotranscriptomes following trimming of low quality (<10) ends and rejection of average quality <20.

Library name *	Strains used **	Total number of sequences	Total number of residues	Average length	Median size	SRA accession
Arg-A	30	38,060,109	10,385,270,028	272.9	300	SRR1168894
Arg-N	30	39,759,543	11,417,825,638	287.2	300	SRR1168938
BolCol-A	21,28,37,40,43,47	38,418,718	10,546,661,014	274.5	300	SRR1168892
BolCol-N	21,28,37,40,43,47	39,223,445	11,258,595,763	287	300	SRR1168893
BolNat-A	24, 50, 54, 56	38,573,948	10,165,647,571	263.5	300	SRR1168890
BolNat-N	24, 50, 54, 56	48,570,803	13,994,680,783	288.1	300	SRR1168891
Chile-A	6	34,269,672	9,102,400,847	265.6	300	SRR1168882
Chile-N	6	38,866,808	11,069,693,781	284.8	300	SRR1168885
Peru-A	4, 5	38,540,450	9,775,095,017	253.6	300	SRR1168888
Peru-N	4, 5	41,292,975	11,889,399,223	287.9	300	SRR1168889
**Total**		395,576,471	109,605,269,665			

*Arg = Argentina. BolCol = Bolivian colony. BolNat = Bolivian F1 strain. The suffix –A stands for adult, -N for nymphal libraries.

**See supplemental table S1 for description of strains.

### cDNA library constructions and next generation sequencing

All 10 SG RNA samples were sent to the Genomic Sciences Laboratory of the North Carolina State University, Raleigh, NC, USA, for Illumina cDNA library constructions and sequencing. Prior to cDNA library constructions, the RNA quality and concentration were checked with the Agilent Bioanalyzer 2100 using an Agilent RNA 6000 Nano Chip (Agilent Technologies, USA) and 0.5 µg of each cDNA library was used to prepare Illumina cDNA sequencing libraries. Poly-A mRNA was purified using the oligo-dT beads provided in the NEBNExt Poly(A) mRNA Magnetic Isolation Module (New England Biolabs (NEB), USA). Libraries were prepared using the NEBNext Ultra Directional RNA Library Prep Kit (NEB) for Illumina and indexed with the NEBNext Mulitplex Oligos for Illumina (NEB). The Poly-A mRNA was chemically fragmented and primed with random oligos for first strand cDNA synthesis with a heating step of 94°C for 5 min. First strand cDNA synthesis was performed with an incubation time of 10 min at 25°C, 50 min at 42°C, and 15 min at 70°C. Second strand cDNA synthesis was carried out with dUTPs to preserve strand orientation information. The sample was purified, end repaired and dA-tailed prior to adaptor ligation. Illumina Multiplex Adaptors were ligated, the ligation reaction was purified according to the protocol for a 500–700 bp insert, and a PCR amplification of 15 cycles was performed. The PCR product was purified and the cDNA libraries were checked for quality and concentration with the Agilent Bioanalyzer 2100 using a High Sensitivity DNA Chip (Agilent Technologies, USA). The 10 prepared Illumina cDNA libraries were pooled in equal molar amounts and the resulted, final pooled cDNA library was run on a Illumina MiSeq instrument using the MiSeq Reagent Kit v3 (Illumina, USA). Ten independent 2×300 bp paired-end runs were carried out and clustered at a concentration of 8pM. The software packages Real Time Analysis (RTA), version 1.18.42, MiSeq Control Software (MCS), version 2.3.0, and MiSeq Reporter, version 2.3.32, were used for sequencing and to generate fastq files.

### Bioinformatic analysis

Bioinformatic analyses were conducted following methods described previously [37], [38]. Briefly, the fastq files were trimmed of low quality reads (<10, rejecting those that have an average <20) and concatenated for single-ended assembly using the Abyss [39] and Soapdenovo Trans (http://arxiv.org/ftp/arxiv/papers/1305/1305.6760.pdf) assemblers using k parameters from 21–91 in 10 fold increments. The combined fasta files were further assembled using a iterative blast and cap3 pipeline as previously described [40]. Coding sequences were extracted based on the existence of a signal peptide in the longer open reading frame (ORF) and by similarities to other proteins found in the Refseq invertebrate database from the National Center for Biotechnology Information (NCBI) as well as proteins from Hemiptera deposited at NCBI's GenBank and from Swissprot. CDS were automatically annotated based on a program written by JMCR that searched a vocabulary of ∼300 words on the matches of the Swissp and Refseq databases, as well as the CDD, KOG and PFAM databases. This automatic annotation was further refined by manual annotation when needed. Transposable elements were discovered by RPSBlast of the transcripts against a PSIBLAST-made database derived from the clusterization at 90% identity on 50% of the larger sequence length of all larger open reading frames available in the Repbase database [41]. An e value of 1e-15 was considered as a threshold call. Reads for each library were mapped on the deducted coding sequences using blastn with a word size of 25, 1 gap allowed and 96% identity or better required. Up to five matches were allowed if the scores were the same as the largest score. A *Χ* ^2^ test was performed for each CDS to detect statistically significant differences between the number of reads in paired comparisons. The results of these tests are mapped to the hyperlinked excel sheet presented as a supplemental file S1. The normalized ratio of the reads were calculated as r1×R2/[R1×(r2+1)] and r2×R1/[R2×(r1+1)] where r1 and r2 are reads for libraries 1 and 2, and R1 and R2 are total number of reads from libraries 1 and 2 mapped to all CDS. One was added to the number of reads in the denominator to avoid division by zero. Sequence alignments were done with the ClustalX software package [42]. Phylogenetic analysis and statistical neighbor-joining bootstrap tests of the phylogenies were done with the Mega 6.0 package [32]. For visualization of synonymous and non-synonymous sites within coding sequences, the tool BWA aln [43] was used to map the reads to the coding sequences (CDS), producing SAI files that were joined by BWA samse which was converted to BAM format and sorted. The samtools package [44] was used to do the mpileup of the reads (samtools mpileup) and the bcftools program from the same package was used to make the final variant call format (VCF) file containing the single nucleotide polymorphic sites (SNP), which were only taken if the CDS coverage depth was at least 20 and the quality was 13 or better (default). Determination of whether the SNP lead to a synonymous or non-synonymous codon change was achieved by a program written in Visual Basic by JMCR, the results of which are mapped into the excel spreadsheet shown as supplemental file S1. Heat maps were made with the gplots and heatmap.2 programs [45] from the R package [46] using average normalized data (row values were divided by the row average).

### Data accession

The raw RNAseq data were submitted to the Sequence Read Archive (SRA) of the NCBI under bioproject PRJNA238208. The raw data file accession numbers can be found in Table 1. Extracted coding sequences were submitted to the Transcriptome Shotgun Annotation (TSA) portal of the NCBI under accessions GBBI01000001-GBBI01005114.

## Results/Discussion

### Selection of *T. infestans* strains for next generation sequencing

To detect the most genetic variations in the transcriptome of different *T. infestans* populations from South America, especially between the Bolivian *T. infestans* strains either kept for several years in our laboratory or collected recently in the field (F1 generation), the haplotypes of 22 *T. infestans* strains (Supplemental table S1) were identified. *T. infestans* strains with different haplotypes were selected for Illumina sequencing.

A total of 44 sequences of the ITS-1, 5.8S and ITS-2 were obtained from sylvatic, peridomestic and domestic *T. infestans* specimens. The sequence alignment revealed the existence of only two combined haplotypes (CH) named T.inf-CH1A and T.inf-CH2A (Table 2). Their length and AT content were of 1304 bp and 1310 bp and 67.41% and 67.56%, respectively. The sequences of the haplotypes T.inf-CH1A and T.inf-CH2A were identical to those found in two different ITS1-5.8-ITS2 CH regions previously reported for *T. infestans* [4]. The 5.8S gene had a length of 155 bp and an AT content of 41.94% and was identical in all specimens analysed. The relation between geographic origins and genetic characteristics of the populations, whether sylvatic, peridomestic or domestic, suggests that CH1A is present only in Bolivia and Peru and well-established among peridomestic, domestic and sylvatic samples, while CH2A is only found in domestic and peridomestic habitats of Chile and Argentina and in one locality from Bolivia (Table 3). In the 481 bp-long alignment, including the two ITS-2 haplotypes, representing the samples of the *T. infestans* analysed and the eleven ITS-2 sequences from GenBank, 13 variable positions appeared (2.70%) of which, 3 were substitutions (0.62%), including 2 parsimony-informative positions (P-info) and 1 singleton site, and 10 (2.05%) gapped positions. The analysis of variable positions among all ITS-2 sequences, revealed the existence of only 8 different haplotypes for *T. infestans*, although there is only the complete sequence of five of them (Table 3). In the 703 bp-long alignment including the only one ITS-1 haplotype representing the samples of the *T. infestans* analysed and the six ITS-1 sequences from GenBank, 36 variable positions appeared (5.12%) of which, 2 were substitutions (0.28%), including 1 P-info position and 1 singleton site, and 34 (4.84%) gapped positions. The main genetic variation was observed in the minisatellite region between positions 179 and 217 in the alignment (Table 3). As in the case of the ITS-2, six different ITS-1 haplotypes have been described for *T. infestans*, although three of them are partial or incomplete sequences.

**Table 2:** Haplotype distribution of 44 *T. infestans* specimens analysed according to their geographical origin and ecotope.

Haplotype code	Bolivia			Peru	Chile	Argentina
	Domestic	Peridomestic	Sylvatic	Peridomestic/domestic	Domestic	Peridomestic
T.inf CH1A	8	18	4	4	-	-
T.inf CH2A	2	4	-	-	2	2

**Table 3:** Polymorphic sites allowing differences between ITS-2 and ITS1 rDNA haplotypes of *T. infestans* samples analysed from Bolivia, Peru, Chile and Argentina and those available in GenBank.

ITS-2		
GenBank Acc. No.	Haplotype code	Variable positions
		222 2334444444 4444444444 4444444444 444444444
		5557777222 2454444555 5555555666 6666666777 777777788
		4675678678 9726789012 3456789012 3456789012 345678901
AJ576051	T.inf-GT1	T------AAA TCAATATTTA AAAAGAAAAG AGATGCGCAA TCATTTTTT
AJ576051	T.inf-H1 *	.................................................
AJ576054	T.inf-GT2	A—ATAT.....T....................................
AJ576054	T.inf-H2 *	A—ATAT.....T....................................
AJ576052	T.inf-GT3	AATATAT.....T....................................
AJ576053	T.inf-GT4	A—AT----- -.......................................
AJ576055	T.inf-GT5	A—ATAT...........................................
HQ333211	T.inf-ITS2Hap1	.------....................--- ---------- ---------
HQ333212	T.inf-ITS2Hap2	A—AT—....................--- ---------- ---------
HQ333213	T.inf-ITS2Hap3	A—AT—....T...............--- ---------- ---------
HQ333214	T.inf-ITS2Hap4	A—ATAT.....T..............--- ---------- ---------
AY860387	ITInf72	AATATAT.....T....................................
AY860388	ITInf74	A------......------- ---------- ---------- ---------

ITS-1		
GenBank Acc. No.	Haplotype code	Variable positions
		1111111111 1112222222 2222222222 222335
		2667999999 9990000000 0001111111 113191
		3459123456 7890123456 7890123456 783769
AJ576051	T.inf-GTA	-TA------- ---------- ---------- -AAGA-
AJ576051	T.inf-HA *	.....................................
AJ582024	T.inf-GTB	---------T AAAATAAAAA GCCGCAAAGA CC.-.-
AJ582025	T.inf-GTC	-..-ATAAAT AAAATAAAAA GCCGCAAAGA CC.-.-
HQ437705	T.inf-HD	-..------- ---------- ---------- -....A
HQ437706	T.inf-HE	-..A------ ---------- ---------- -....-
DQ118960	TiITS10037	T..------- ---------- ---------- -.-.GA

*haplotypes from present paper; variable positions = numbers (to be read in vertical) refer to variable positions obtained in the ITS-2 and ITS-1 alignments obtained with MEGA 6.0; symbols: identical positions = .; indel = -; indel position in 3′ end of the alignment = not sequenced. Haplotypes that seem to be identical: T.inf-GT1 = T.inf-ITS2Hap1; T.inf-GT2 = T.inf-ITS2Hap4; T.inf-GT3 = ITInf72.

Following the above analysis, and having in consideration the determination of developmental stage and strain specific variations in the *T. infestans* salivary gland transcriptome, we selected 14 of the 22 available strains of *T. infestans* (Supplemental Table S1) as follows (strain numbers refer to strain named in the first column of supplemental table S1): All Peruvian, domestic and peridomestic (strains 4 and 5), Chilean, 1 domestic strain (6), and Argentinian, 1 peridomestic strain (30); from the more numerous Bolivian strains the two T.inf-CH2A haplotype strains covering the two different regions in the Department of Santa Cruz in Bolivia (Cabezas and Boyuibe, F1 generation, domestic and peridomestic strains 50 and 54) were chosen plus two sylvatic (colony strains 43 and 47), four peridomestic (colony strains 28, 40 and F1 generation strains 24 and 56) and two domestic *T. infestans* strains with the T.inf-CH1A haplotypes (colony strains 21 and 37), as detailed in supplemental table S1 and table 1.

### Overall characteristics of the sialome of *T. infestans*

From the fourteen *T. infestans* strains selected for next generation sequencing, ten Illumina libraries were prepared and sequenced discriminating nymphal (5 libraries) and adult *T. infestans* transcripts (5 libraries) of different triatomine populations from Chile, Peru, Argentina and Bolivia, including two Bolivian libraries, one from colony strains and another from insects recently collected in the field (F1 generation) (Supplemental table S1 and Table 1). A total of over 395 million sequences, summing over 109 billion nucleotides, were recovered from the 10 sequenced libraries, ranging from 38 to 48 million sequences, or reads, per library (Table 1). The reads had an average length of 277 bases and a median of 300 bases after clipping low quality (<10) bases. The assembly allowed the extraction of 11,188 coding sequences (CDS) having an average deducted protein length of 398 amino acids. These CDS mapped a total of 213,756,622 reads from all 10 libraries, or about half of the total reads (Table 4). Following an automated classification, the CDS were classified into putative Secreted, Housekeeping, Transposable element, Viral and Unknown (Table 4). Although the 2,228 CDS of the Secreted class were only 20% of the total 11,188, they accrued 49% of the reads. On average these CDS were assembled each from 46 thousand reads. On the other hand, the Housekeeping class represented 65% of all CDS, collected 49% of the reads, and was assembled with an average of 14 thousand reads per CDS. Transposable element-coding sequences were represented by 5% of the CDS, a larger value than found on previous sialotranscriptomes, which is near 1%. Among the viral sequences identified, the assembly recovered the 5,388 nucleotides of the full length CDS for the nonstructural protein precursor of the *Triatoma* virus [47], which was assembled from over 171 thousand reads. Other viral fragments were additionally found, some of which may derive from retrotransposable elements.

**Table 4:** Classification and metrics of the assembled sialome of *Triatoma infestans*.

Class	Number of CDS *	Number of Reads	Reads/CDS	Percent total CDS	Percent total reads
Secreted	2,228	104,622,738	46,958	19.91	48.94
Housekeeping	7,281	103,850,665	14,263	65.08	48.58
Transposable element	560	2,092,706	3,737	5.01	0.98
Viral	47	990,621	21,077	0.42	0.46
Unknown	1,072	2,199,892	2,052	9.58	1.03
Total	11,188	213,756,622		100	100

*CDS = Coding sequence.

A description of the sialome of *T. infestans* was disclosed in 2008 based on 1,534 EST sequences sequenced by Sanger methodology [6]. From this work, 167 protein sequences have been deposited to GenBank, including many lipocalin sequences, which are abundant in triatomine sialomes [48], salivary enzymes such as apyrase, and other triatomine-specific proteins. The current update has identified over 1 order of magnitude additional sequences that appear as full length, or near full length, 5,114 of which have been deposited to GenBank. This submission expands the *T. infestans* sialome database and should help in the identification of pharmacologically active proteins as well as markers of vector exposure. Presently we will describe highlights of this expanded transcriptome, identify and describe stage specific and geographic specific protein variants, and attempt a measure of the polymorphism of the *T. infestans* sialome. This work has no biological replicates in the comparisons described below, a fact considered in this discussion.

### Highlights of the expanded *T. infestans* sialotranscriptome

Compared to classical Sanger-derived transcriptomes with a few thousand sequenced EST's, next-generation Illumina libraries derived from hundreds of millions of sequences provides for an unprecedented depth of transcript coverage allowing detection of poorly expressed mRNA as well as their quantitation as hundreds, or millions of reads accrue to each assembled contig. For example, the most expressed CDS in this sialome is for the peptide trialysin, which accrued over 13 million reads and deriving an RPKM = 77,477 when all reads from all libraries are considered. Supplemental file S2 presents a classified version of supplemental file S1 that has the different functional classes sorted by their overall RPKM, allowing for fast identification of well-expressed CDS, including putative secreted proteins. In the following, we present some remarkable findings in this sialotranscriptome.

#### Enzymes

Serine proteases have been described earlier in *T. infestans* salivary homogenates [49], and one such enzyme has been found in a previous sialotranscriptome (gi|149689028) [6]. The current sialotranscriptome identified this enzyme coded by TiSigP-124791, with an RPKM = 1,243 plus two other full length enzymes that have an RPKM>700, plus several others with lower expression. TiSigP-124931 is 76% identical with a previously described *Panstrongylus megistus* salivary trypsin [50]. A salivary metalloprotease was found well expressed (RPKM = 715), being 92% identical to a protein deducted from the *T. matogrossensis* sialotranscriptome [51]. An asparaginyl peptidase and a Cathepsin L were also found well expressed, with RPKM>790. Apyrases are enzymes that hydrolyze ATP and ADP, mediators of platelet and neutrophil aggregation, and were found to be members of the 5′ nucleotidase family in *T. infestans* [52]. The current sialotranscriptome identified additional well expressed transcripts being only 66% identical to the described salivary apyrase of *T. infestans*, suggesting that this salivary activity derives from a family of related proteins suggestive of gene duplications, a common motif in salivary genes of blood sucking arthropods [5]. Inositol phosphate phosphatases are also commonly found in the sialotranscriptomes of blood sucking Hemiptera [48]; a few additional representatives of this family were found in the current sialotranscriptome.

#### Protease inhibitors

The Kazal family of protease inhibitors is found expanded and abundantly transcribed in the sialotranscriptome of bloodsucking Hemiptera [48]. Several novel full length representatives were found in the current transcriptome, nine of which have RPKM>650. A pacifastin, 66% similar to a *Rhodnius prolixus* homologue was identified, with a RPKM = 869.

#### Lipocalin superfamily

The lipocalin family of proteins is extremely expanded in triatomines where it exerts anti-clotting functions as well as antagonism of biogenic amines, leukotrienes and other inflammatory mediators and carriers of NO in *Rhodnius* [48], [53]. Since many of the differentially transcribed CDS described further below belong to this superfamily, we aligned the sequences discovered in this transcriptome with lipocalins described from previous triatomine sialotranscriptomes, generating a phylogenetic tree with at least 13 different clades containing *T. infestans* proteins. Of these 13 clades, only 3 have at least one protein that has been studied, including those named Triabin (an anti-clotting protein) [54], [55], Pallidipin (an anti-platelet protein that binds thromboxane A2) [56], [57] and the antigen Procalin [58]. Supplemental figure S1 displays this phylogenetic tree and indicates near the CDS name those that were previously characterized, as well as those that are differentially expressed in nymphs, adults or particular geographic populations. The tree also shows that over 40 novel lipocalin sequences were found in this *T. infestans* sialotranscriptome.

#### Salivary odorant binding proteins

Two members of the odorant binding protein family have been found expressed in the sialotranscriptome of *T. infestans* (gi|149689106 and gi|149689110). Eight additional members were found in the current transcriptome, underlining the gene expansion of this family within *T. infestans*. Two of these novel transcripts are well expressed, with RPKM>1000.

#### Antigen 5 family

Eight full length or near full length novel members of this family were identified, three of which with RPKM>1000. Members of this family expressed in the salivary glands of *Dipetalogaster maxima* were shown to possess superoxide dismutase activity [59].

#### Antimicrobial peptides and lysozyme

Twenty transcripts coding for defensins, diptericin, attacin and lysozyme were found, only one of which has been previously identified. Antimicrobial peptides are commonly found in sialotranscriptomes of blood sucking arthropods [5].

#### Deorphanized and novel putative secreted proteins

Supplemental file S2 identifies 19 CDS similar to previously identified orphan Hemiptera proteins, five of which being well expressed (RPKM>1000), and over 1,000 additional CDS coding for putative secreted proteins not producing a good match to any known protein; however, only 13 accrue a RPKM>500.

### Differentially expressed transcription

**Figure 1:**
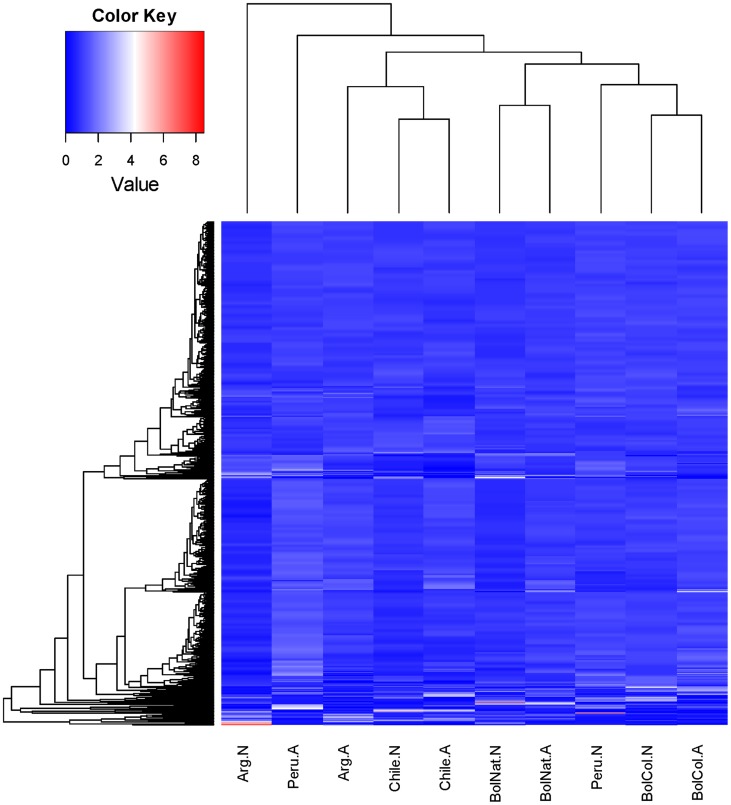
Z score normalized heat map of the RPKM values of the 10 triatomine libraries. Results show only CDS having an overall RPKM equal or greater than 20, totaling 4,207 CDS for the Chilean (Chile), Argentinian (Arg), Peruvian (Peru), Bolivian F1 (BolNat) and Bolivian colony (BolCol)-derived libraries from adults (.A) or nymphal (.N) organisms.

To obtain a general view of the differential transcriptome expression among the 10 libraries, we produced a hierarchical clustering-based heat map of average normalized RPKM values for the contigs with overall RPKM equal or larger than 20, totaling 4,207 CDS (Fig. 1). Notice that following normalization, the average row value is equal to one, with transcripts expressing above average having values above one and vice versa. The resulting clusterization is complex and shows that only a minority of the CDS have 4 or more units above average (represented by white or red color in the graph). Some of these transcript differences will be further highlighted in the following sections.

### Differentially expressed transcripts according to developmental stage

#### Nymphal specific proteins

We combined the number of reads per CDS from all nymphal and all adult libraries allowing detecting nymphal enriched and adult enriched sequences, independent of geographic origin. A total of 180 CDS were significantly 10 fold or more expressed in nymphal than in adult libraries (Table 5, supplemental file S3). Interestingly, among the most nymphal-specific CDS are 43 coding for cuticle proteins, including the homolog of *Acyrthosiphon pisum* cuticular protein CPG12-like precursor, assembled from 1,153 reads from nymphal libraries and zero from adult libraries. Nymphal cuticle proteins are different from the hardier adult cuticle and the genes coding for these CDS are developmentally regulated [60]. Similarly the sequence coding for juvenile hormone (JH) acid methyltransferase was assembled from 1,053 reads from nymphal libraries, and zero from adult libraries. This enzyme is crucial for nymphal developmental processes and is thus expected to be enriched in nymphs. Two sequences coding for JH binding proteins are also overexpressed in nymphs. Three sequences coding for cytochrome P450 enzymes were overexpressed in nymphal libraries and may be related to hormonal regulation or cuticle metabolism. A gene coding for chorion peroxidase may also be related to cuticle metabolism. These CDS probably originate from contaminating tissues during dissection, but the high number of sequences (near half billion) allowed for their assembly.

**Table 5:** Classification of coding sequences at least 10× overexpressed in nymphal libraries when compared to adult libraries.

Class	Average RPKM all adults	SE	Average RPKM all nymphs	SE	Number of CDS
**Secreted**					
Lipocalins	98.1900	59.6835	2,757.7731	1,036.9280	8
Antigen-5 family	62.9588	37.0020	1,346.8399	556.8039	2
Other secreted	4.2330	3.7634	53.1660	40.0030	43
C-type lectin	2.5787	1.7501	31.2737	19.7273	2
Proteases	1.4720	1.0894	31.1334	17.0503	7
Conserved secreted	0.3529	0.0849	13.0157	4.7365	16
Odorant/pheromone-binding proteins	0.9167	0.4061	15.7200	3.7388	2
Protease inhibitor	0.1309	0.0379	2.9161	0.6278	2
**Housekeeping**					
Signal transduction	1.7052	1.0083	73.5966	32.3177	4
Protein modification	1.0268	0.7818	29.8173	22.9426	43
Extracellular matrix	0.4108	0.1552	20.9722	5.7174	2
Transcription factor	0.3172	0.0000	19.8533	0.0000	1
Unknown conserved membrane protein	0.0735	0.0428	15.3980	6.3249	2
Lipid metabolism	0.7016	0.3076	12.5505	2.7891	1
Unknown product	0.8774	0.5600	12.5492	7.7100	3
Immunity	0.1292	0.0448	10.6898	6.1938	11
Transcription machinery	0.2775	0.2224	8.5677	2.8206	1
Protein export	0.1714	0.0000	6.8353	0.0000	3
Oxidant metabolism/Detoxification	0.1940	0.0768	6.1823	2.2717	5
Transporters and channels	0.0953	0.0325	2.7344	0.3883	3
Unknown conserved	0.1298	0.0842	2.5167	1.0910	8
Amino acid metabolism	0.1666	0.0000	2.0941	0.0000	11
Total					180

Among the products coding for secreted proteins overexpressed in nymphal libraries, 8 lipocalins were found, with an average RPKM over 2,500, and being 40 to 80 times more expressed in nymphs. This family of proteins is associated with agonist scavenging (kratagonist) as well as anti-clotting activities [48]. Two members of the antigen 5 family, some of which have been described to have superoxide dismutase activity [59] were also found with increased nymphal expression, with average RPKM of 1,340 (Table 5).

The interested reader can sort the provided Excel spreadsheet (supplemental file S1) in the desired comparison columns to obtain the names and sequences identified above and in the following sections of this manuscript.

#### Adult specific proteins

When adult overexpressed CDS were investigated, only 29 were found 10× more expressed than in the combined nymphal libraries (Table 6, supplemental file S3). These included two coding for vitellogenin, an oocyte storage protein produced by adult females [61], assembled most probably from contaminating fat body. These two CDS were 235 and 607 times more expressed in adult libraries. Another adult-specific marker found was a transcript coding for the doublesex transcription factor, involved in adult sex determination [62]; it was assembled from 248 reads from the adult libraries and 5 from the nymphal libraries, having an RPKM of 2.8 related to the adult libraries. Among the secreted products, a member of the GGY family, related to antimicrobial peptides in *C. elegans* [63] and homologous to *Rhodnius prolixus* salivary proteins named for their Gly-Gly-Tyr repeats [64] was found 10× overexpressed in adult libraries (Table 6). Transcripts coding for two lipocalins were 13 and 10.6 times overexpressed in adults, and their average RPKM was over 10,000.

**Table 6:** Classification of coding sequences at least 10× overexpressed in adult libraries when compared to nymphal libraries.

Class	Average RPKM all adults	SE	Average RPKM all nymphs	SE	Number of CDS
**Secreted**					
GGY family	12.8804		1.2140		1
Lipocalin	10,298.1195	2,389.6833	836.9520	131.5580	2
Other secreted	3.7156	1.4266	0.2025	0.1176	11
**Housekeeping**					
Detoxification	10.5979		0.8699		1
Extracellular matrix	7.3566	4.4562	0.6073	0.4061	2
Amino acid metabolism	8.0896		0.7302		1
Lipid metabolism	1.6272		0.1415		1
Protein synthesis machinery	0.4646		0.0000		1
Signal transduction	1.4232		0.1047		1
Storage	75.6115	51.1812	0.1265	0.0817	2
Transcription factor	2.8275		0.0548		1
Transcription machinery	0.6043		0.0439		1
Unknown conserved	1.8744		0.1093		1
Unknown product	21.1070	14.3150	1.4621	1.0338	2
**Transposable element**	20.1144		0.5998		1
Total					29

It has been previously reported in *R. prolixus* that members of the nitrophorin family are progressively expressed from the first instar nymph to the last and in adults [65]. This developmental control of gene expression is suggestive of epigenetic regulation [66]–[68] involving DNA-modifying as well as histone-modifying enzymes. We have identified 42 transcripts in the *T. infestans* sialotranscriptome that might be involved in epigenetic regulation, including several full length histone-lysine-N-methyltrasferases, histone deacetylase complex members and sirtuins. All have similar expression among nymphal and adult libraries, with RPKM varying between 2 and 470. Identification of these gene products can help the design of RNAi experiments to evaluate epigenetic regulation of gene expression in *T. infestans*.

### Differentially expressed transcripts according to geographical origin

For these analyses, nymphal and adult reads from each geographical area were combined and compared with the sum of reads from the other libraries, to identify differentially expressed CDS specific to each region. We report here only those CDS that are significantly expressed 10× or more according to these comparisons.

#### Argentinian overexpressed transcripts

Twenty four transcripts were found 10 fold or more overexpressed in Argentinian bugs (Table 7, supplemental file S3). Six lipocalins with high expression levels include Ti-133850 (RPKM = 8,622, other libraries RPKM = 16.5), one Kazal-domain containing peptide and 4 novel putative secreted proteins constituting gene products coding for candidate secreted proteins overexpressed in Argentinian *Triatoma* (Table 7). Transposable elements were also identified. One highly expressed contig of unknown class (Ti-123232, RPKM = 13,318) codes for a protein that is 36–46% identical to three *Ixodes scapularis* proteins and has no other significant matches to the NR database; the remainder libraries account for a small RPKM = 1.65. Interestingly, the Argentinian population also differentially expresses a member of the cytochrome c oxidase polypeptide IV (RPKM = 38.4, others = 0.17), which is a nuclearly encoded mitochondrial gene. A second isoform of this enzyme is also found in the database, but it is not differentially expressed among the populations (TiSigP-123830), and has a higher RPKM in the Argentinian population of 378, while all other populations it is 405.

**Table 7:** Transcripts found overexpressed (>10 fold) on Argentinian population.

Class	Average RPKM Argentina	SE	Average RPKM Others	SE	Number of CDS
**Secreted**					
Lipocalins	14,469.44	5,166.37	984.79	504.35	6
Kazal peptide	262.67		11.79		1
Other secreted	13.42	6.57	0.53	0.29	4
**Housekeeping**					
Metabolism	22.05	6.98	0.92	0.39	3
Protein export	4,220.34		137.56		1
Signal transduction	9,882.16		158.08		1
Transporters	20.41		0.89		1
Unknown conserved	4,341.99	3,026.38	3.44	1.26	2
Unknown	12.27		0.99	0.00	1
**Transposable element**	566.69	472.83	26.43	22.30	4
Total					24

#### Bolivian colony overexpressed transcripts

Only 10 transcripts were found 10 fold or more overexpressed in the Bolivian colony, all with relatively low transcription levels, the maximum RPKM being 20 (Table 8, supplemental file S3). Among these a viral transcript was found best matching a structural protein precursor of *Drosophila A* virus, with 26% identity and 45% similarity over 442 amino acids. It has an RPKM of 7.1 in the Bolivian library and only 0.0097 in the combined remaining libraries.

**Table 8:** Transcripts found overexpressed (>10 fold) on Bolivian colony population.

Class	Average RPKM Bolivian colony	SE	Average RPKM Others	SE	Number of CDS
**Secreted**					
Lysozyme	5.72		0.45		1
Other secreted	10.44	3.49	0.71	0.33	2
**Housekeeping**					
Extracellular matrix	3.55	0.50	0.30	0.03	2
Transporters	1.87		0.15		1
Signal transduction	20.07	12.14	1.38	0.87	2
**Transposable element**	11.47		0.65		1
**Viral**	7.11		0.01		1
Total					10

#### Bolivian F1 overexpressed transcripts

On the other hand, when the Bolivian F1 population was analyzed for overexpressed transcripts, 30 were found, including 15 lipocalins, several of which appear well expressed (Table 9, supplemental file S3). Among these, Ti-130968 has a RPKM of 2,888 from the Bolivian F1 reads while all other reads amount to a value of only 5.26. Similarly, TiSigP-123883 has a high expression value (RPKM = 18,053) deriving from the Bolivian F1 library but only 59.3 from the remainder libraries.

**Table 9:** Transcripts found overexpressed (>10 fold) on Bolivian F1 population.

Class	Average RPKM Bolivian F1	SE	Average RPKM Others	SE	Number of CDS
**Secreted**					
Lipocalins	3,913.92	1,135.50	111.38	46.76	15
Other secreted	7.77	3.50	0.52	0.33	3
**Housekeeping**					
Extracellular matrix	4.97	1.66	0.36	0.16	3
Metabolism	955.37		18.81		1
Protein export	454.62		44.53		1
Signal transduction	1.46		0.00		1
Storage	5.76		0.36		1
Transporters	2.03		0.01		1
Unknown conserved	2.95		0.00		1
Unknown	5.50	0.35	0.38	0.00	2
**Transposable element**	6.73		0.65		1
Total					30

#### Chilean overexpressed transcripts

Three highly expressed lipocalins and one relatively high expressed Kazal peptide (RPKM = 1,012) are overexpressed in the Chilean population, among a total of 14 transcripts (Table 10, supplemental file S3). The lipocalin TiSigP-128283 appears over 60 fold overexpressed, with a RPKM = 2,323.

**Table 10:** Transcripts found overexpressed (>10 fold) on Chilean population.

Class	Average RPKM Chile	SE	Average RPKM Others	SE	Number of CDS
**Secreted**					
Lipocalins	2,179.35	345.93	41.75	4.64	3
Kazal peptide	1,012.43	0.00	44.95	0.00	1
Other secreted	3.14	0.00	0.28	0.00	1
**Housekeeping**					
Extracellular matrix	4.56	1.77	0.31	0.11	2
Nuclear regulation	2.65	0.00	0.22	0.00	1
Transporters	261.25	0.00	16.48	0.00	1
Unknown conserved	47.77	0.00	1.81	0.00	1
Unknown	4.90	0.00	0.13	0.00	1
**Transposable element**	21.26	13.90	1.93	1.33	3
Total					14

#### Peruvian overexpressed transcripts

Thirty nine transcripts were found 10 fold or more overexpressed in the Peruvian library, including three highly expressed lipocalins and 10 other putative secreted peptides of low expression (Table 11, supplemental file S3). Of note are several viral transcripts of moderate to high RPKM values (ranging from 38–3,810), but significantly higher expressed in Peruvian bugs, being so over 1,000 fold overexpressed. This set includes Ti-133027 and Ti-126254 which are 26–36% identical and 41–52% similar to the *Drosophila A* virus mentioned above, but are not the same contigs described above in the Bolivian colony section. Ti-130944 and Ti-141814 both best match the Sugarcane yellow leaf virus TNA-directed RNA polymerase, being 30% identical and 46% similar over a stretch of 413 amino acids. Some of the eight transcripts of the unknown class produce matches to viral proteins, albeit with an e value higher than 1e-15 and could also derive from viral products.

**Table 11:** Transcripts found overexpressed (>10 fold) on Peruvian population.

Class	Average RPKM Peruvian	SE	Average RPKM Others	SE	Number of CDS
**Secreted**					
Lipocalins	21,025.36	8,304.96	1,100.73	233.84	3
Secreted, conserved	159.85	0.00	15.67	0.00	1
Other secreted	10.71	3.97	0.75	0.32	9
**Housekeeping**					
Extracellular matrix	5.84	0.00	0.36	0.00	1
Metabolism	2.14	0.00	0.02	0.00	1
Protein modification	3.64	0.00	0.23	0.00	1
Protein synthesis	4.67	0.00	0.07	0.00	1
Signal transduction	2.17	0.00	0.19	0.00	1
Storage	309.86	0.00	14.57	0.00	1
Transcription machinery	15.41	0.00	0.00	0.00	1
Unknown conserved	7.07	3.13	0.34	0.08	2
Unknown	531.22	260.82	24.91	13.88	8
**Transposable element**	17.46	2.63	1.21	0.35	5
**Viral**	982.54	787.76	53.30	46.14	4
Total					39

### Salivary gland polymorphism

Single nucleotide polymorphisms were identified from each of the 10 libraries and mapped into the derived CDS. A rich text format (RTF) file is provided for each CDS showing the polymorphisms, hyperlinked to the Excel supplemental file S1. Of the 11,188 deducted CDS, one or more polymorphic sites were identified in 5,391 when all data was combined (Table 12). The classes Viral, Transposable element, Unknown and secreted had the highest percentages of both synonymous (S) and non-synonymous (NS) polymorphisms, and also displayed high NS/S ratios, although the highest ratio derived from the protein synthesis category (Table 12). Sixty three members of the lipocalin family had on average 2.5 NS polymorphisms per 100 codons while the secreted class rate was 0.83 S/100 codons (results not shown). This high rate of NS to S rate suggests that these genes are under positive selection, as has been suggested for a subset of salivary genes of the mosquito *Anopheles gambiae* [7].

**Table 12:** Polymorphisms detected on the *Triatoma infestans* sialotranscriptome according to functional class.

Class	Average Synonymous *	SE	Average Non- synonymous *	SE	NS/S	N
Extracellular matrix	1.047	0.092	0.484	0.053	0.462	180
Transporters	0.594	0.049	0.419	0.048	0.704	191
Signal transduction	0.555	0.028	0.406	0.03	0.731	677
Protein export	0.433	0.047	0.332	0.052	0.766	132
Protein modification	0.619	0.08	0.53	0.068	0.857	130
Detoxification	1.009	0.119	0.872	0.122	0.865	100
Cytoskeletal	0.511	0.051	0.451	0.056	0.882	139
Transcription factors	0.721	0.076	0.649	0.123	0.9	113
Nuclear export	0.098	0.023	0.096	0.023	0.976	18
Metabolism	0.669	0.043	0.666	0.051	0.995	496
Proteasome machinery	0.474	0.066	0.498	0.087	1.051	103
Unknown conserved	0.756	0.037	0.84	0.048	1.111	829
Transcription machinery	0.637	0.049	0.712	0.077	1.118	354
Nuclear regulation	0.875	0.094	0.98	0.096	1.12	277
Immunity	0.658	0.103	0.764	0.201	1.161	45
Viral	1.66	0.293	2.044	0.401	1.231	34
Storage	0.453	0.145	0.569	0.241	1.256	9
Transposable element	1.385	0.076	2.206	0.095	1.594	447
Unknown	1.223	0.098	2.229	0.135	1.823	306
Secreted	0.938	0.046	1.942	0.093	2.07	702
Protein synthesis	0.382	0.056	0.823	0.116	2.158	109

*Number of synonymous or non-synonymous polymorphisms per 100 codons.

**Figure 2:**
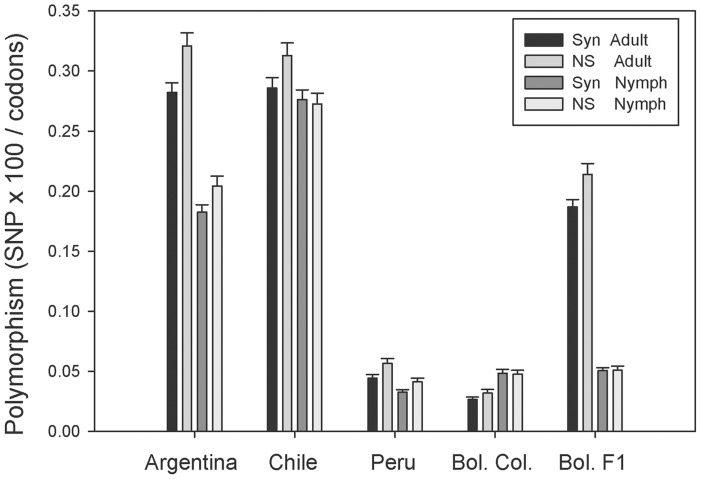
Polymorphism values derived from adult and nymphal coding sequences from different colonies. For each colony (Argentina, Chile, Peru, Bolivian colony and Bolivian F1) synonymous and non-synonymous single nucleotide polymorphisms were determined as indicated in the methods section. The bars represent the average and SE of 5,391 polymorphic CDS.

The degree of polymorphism was also compared among the ten sequenced libraries using the 5,391 polymorphic CDS mentioned above (Fig. 2). Results indicate that polymorphisms are lower in the Peruvian and Bolivian colony libraries, but somewhat surprisingly the nymphal Argentinian library has significant less polymorphism than the adult counterpart, as is the case of the Bolivian F1 library. The Chilean strain that was collected in 1979 has higher polymorphism than the Peruvian strains collected 30 years later, or the recently collected (2012) Bolivian strains. The observed differences do not appear to derive from CDS coverage depth differences between libraries, because the average RPKM for each library were not significantly different, the minimum being 49.1±4.7 (average ± SE) for the Bolivian adult colony and the maximum being 60.5±8.51 for the Peruvian adult library, considering the 5,391 CDS. The differences here observed between nymphs and adults as well as from different geographical areas are congruent to previous *T. infestans* salivary immunological studies [23]. The polymorphism differences between long colonized and more recently colonized insects indicate that colonization time not necessarily leads to reduced polymorphism, which may be maintained by the immune pressure created by using and reusing live animals for colony maintenance.

### Conclusions

Discovery-driven research, as opposed to conventional hypothesis-driven work, should provide or enlarge the knowledge platform to allow further development of hypothesis-driven research. This study enlarges the sialome database for *T. infestans* and at the same time provides information on differentially expressed transcripts based on developmental stage and geographical origin. As a result, 5,114 sequences are now publicly available from GenBank and should be of value in assisting proteomic experiments attempting to determine proteins that might be used to immunologically identify *T. infestans* exposure, as proposed in [23], as well as identification of pharmacologically active salivary proteins of *T. infestans*. Polymorphisms were mapped to individual coding sequences and its differential distribution was identified among geographical strains and developmental stages. This should be of help in developing specific markers of exposure, as well as to avoid extreme polymorphic proteins. Several novel viral and transposon sequences were discovered, which may stimulate virologists or scientists interested in transposable elements to describe new insect viruses or transposable elements. The awkward finding of a second cytochrome oxidase IV gene product in the Argentian population may represent a second horizontal transfer of this gene from a bacterial or mitochondrial genome and remains to be investigated.

Despite the lack of biological replicates in our study, the observed increased sequences associated to cuticle proteins and JH metabolism in nymphs, as well as the increased vitellogenin and doublesex transcription factor in adults which are expected from the known insect physiology, serve to validate the broader differences in transcript abundance found among the different libraries despite lack of replicates, but these should be verified in future follow up work.

## Supporting Information

Figure S1 Bootstrapped phylogenetic tree of triatomine lipocalins. The sequences discovered in this work are represented by Ti- or TiSigp- followed by the contig number. Other sequences were derived from GenBank and are indicated by the first three letters of their genus name, followed by the first three letters of their species name followed by their gi| accession number. Triatoma infestans sequences found in this study are marked with a red symbol. Those from GenBank are marked with a blue symbol. The tree was built from a ClustalX alignment using the NJ algorithm from the Mega package following 1,000 bootstrap iterations. The numbers at the branches represent the bootstrap percentage support when larger than 50.
(RTF)

Table S1 Origin of the *T. infestans* strains from South America, total SG RNA samples prepared for next generation sequencing and haplotypes obtained for the complete intergenic rDNA region (ITS-1, 5.8S, ITS-2).
(DOC)

Table S2 Development times of the different developmental stages of *T. infestans* and numbers of triatomines used in feeding experiments.
(DOCX)

File S1 Excel spreadsheet reporting 11,188 coding sequences (CDS) hyperlinked to various databases, and mapped reads and polymorphism from 10 different libraries. This file should be used for sorting on appropriate fields to identify over/under expressed CDS. Alternatively, Open Office can be used and can be freely downloaded from http://www.openoffice.org/download/. For the hyperlinks to work, make sure local settings allow hyperlinks from exon.niaid.nih.gov to be accessed. http://exon.niaid.nih.gov/transcriptome/T_infestans/T_infestans-S1.xlsx
(DOCX)

File S2 Same as S1, but with CDS functionally sorted. http://exon.niaid.nih.gov/transcriptome/T_infestans/T_infestans-S2.xlsx
(DOCX)

File S3 Detailed data from tables 5–11.
(XLSX)
