# Supplementary material for: An Updated Insight into the Sialotranscriptome of Triatoma infestans: Developmental Stage and Geographic Variations
Source: PLoS Negl Trop Dis. 2014 Dec 4;8(12):e3372. doi: 10.1371/journal.pntd.0003372 (PMC4256203; doi:10.1371/journal.pntd.0003372)
Supplement: Figure S1 — Bootstrapped phylogenetic tree of triatomine lipocalins. The sequences discovered in this work are represented by Ti- or TiSigp- followed by the contig number. Other sequences were derived from GenBank and are indicated by the first three letters of their genus name, followed by the first three letters of their species name followed by their gi| accession number. Triatoma infestans sequences found in this study are marked with a red symbol. Those from GenBank are marked with a blue symbol. The tree was built from a ClustalX alignment using the NJ algorithm from the Mega package following 1,000 bootstrap iterations. The numbers at the branches represent the bootstrap percentage support when larger than 50. (RTF) [file pntd.0003372.s001.rtf]

Supplemental figure S1: Bootstrapped phylogenetic tree of triatomine lipocalins. The sequences discovered in this work are represented by Ti- or TiSigp- followed by the contig number. Other sequences were derived from GenBank and are indicated by the first three letters of their genus name, followed by the first three letters of their species name followed by their gi| accession number. Triatoma infestans sequences found in this study are marked with a red symbol. Those from GenBank are marked with a blue symbol. The tree was built from a ClustalX alignment using the NJ algorithm from the Mega package following 1,000 bootstrap iterations. The numbers at the branches represent the bootstrap percentage support.  Values smaller than 50 are not shown.
