# Supplementary material for: An Updated Insight into the Sialotranscriptome of Triatoma infestans: Developmental Stage and Geographic Variations
Source: PLoS Negl Trop Dis. 2014 Dec 4;8(12):e3372. doi: 10.1371/journal.pntd.0003372 (PMC4256203; doi:10.1371/journal.pntd.0003372)
Supplement: Table S1 — Origin of the T. infestans strains from South America, total SG RNA samples prepared for next generation sequencing and haplotypes obtained for the complete intergenic rDNA region (ITS-1, 5.8S, ITS-2). (DOC) [file pntd.0003372.s002.doc]

Supplemental Table S1. Origin of the *T. infestans* strains from South America, total SG RNA samples prepared for next generation sequencing and haplotypes obtained for the complete intergenic rDNA region (ITS-1, 5.8S, ITS-2).

| *T. infestans*  strain | Country | Department/ province | Municipality/community | Habitat | GPS data | Collection date | Laboratory animal for feeding | RNA sample | ITS1-5.8S-ITS2 haplotype |
| --- | --- | --- | --- | --- | --- | --- | --- | --- | --- |
| 4 | Peru | Arequipa/ Arequipa | Mariano Melgar/ Mariano Melgar | Domestic and peri-domestic | 16°24' 2.2"S  71°29'59.1"W  2464 m | 2008 | Guinea pig | Peru-A  Peru-N | T.inf-CH1A |
| 5 | Peru | Arequipa/ Arequipa | La Joya/ Villa La Joya | Domestic and peri-domestic | 16°25' 40.8"S  71°49'10.7"W  1609 m | 2008 | Rabbit | Peru-A  Peru-N | T.inf-CH1A |
| 6 | Chile | Atacama/ Huasco | Vallenar/ Cachiyuyo | Domestic | 29°02'11.5'' S  70°53'55.1'' W  829 m | 1979 | Guinea pig | Chile-A  Chile-N | T.inf-CH2A |
| 9 | Bolivia | Cochabamba/ Quillacollo | Sipe Sipe/ Sique- Siquia | Peri-domestic | 17°26'22.1''S  66°22'49.4''W  2,622 m | 2012 | - | - | - |
| 12 | Bolivia | Cochabamba/ Capinota | Lipez/ Lipez city | Peri-domestic | 17°34′21.4''S  66°15′52.9″W  2525m | 2007 | - | - | - |
| 15 | Bolivia | Cochabamba/ Campero | Aiquile/ Negro Puyo | Peri-domestic | 18°4′18.2''S  65°9′12.5″W  2192m | 2012 | - | - | - |
| 21 | Bolivia | Cochabamba/ Quillacollo | Sipe Sipe/ Sipe Sipe city | Domestic | 17°25′53.4''S  66°9′25.7″W  2555m | 2007 | Guinea pig | BolCol-A  BolCol-N | T.inf-CH1A |
| 24 | Bolivia | Cochabamba/ Campero | Aiquile/ Negro Puyo | Peri-domestic | 18°4′18.1''S  65°9′12.5″W  2192m | 2012 | Guinea pig | BolNat-A  BolNat-N | T.inf-CH1A |
| 28 | Bolivia | Cochabamba/Esteban Arze | Arbieto/ Arpita | Peri-domestic | 17°34'4.7"S,  66°4'20.8"W  2718m | 2007 | Guinea pig | BolCol-A  BolCol-N | T.inf-CH1A |
| 30 | Argentina | Libertador General San Martín/ Chaco | Pampa del Indio/ Pampa del Indio | Peri-domestic | 26°02’56.5‘‘S  59°56’32.4‘‘W  97m | 2007 | Rabbit | Arg-A  Arg-N | T.inf-CH2A |
| 37 | Bolivia | Cochabamba/ Cercado | Cochabamba/ Cochabamba city | Domestic | 17°25'5.2''S  66°9'32.6''W  2055 m | 2003 | Rabbit | BolCol-A  BolCol-N | T.inf-CH1A |
| 40 | Bolivia | Cochabamba/ Quillacollo | Sipe Sipe/ Sipe Sipe city | Peri-domestic | 17°25′53.4''S  66°9′25.7″W  2555m | 2007 | Rabbit | BolCol-A  BolCol-N | T.inf-CH1A |
| 43 | Bolivia | Cochabamba/ Esteban Arze | La Vina/ Pasacaya | Sylvatic | 17°57'45.3''S  65°51'41.7''W  2.123 m | 2004 | Rabbit | BolCol-A  BolCol-N | T.inf-CH1A |
| 47 | Bolivia | Cochabamba/ Esteban Arze | La Vina/ Pampa Soyco | Sylvatic | 17°58'34.5''S  65°49'29.8''W  2.019 m | 2004 | Guinea pig | BolCol-A  BolCol-N | T.inf-CH1A |
| 50 | Bolivia | Santa Cruz/ Cordillera | Cabezas/ Brecha 10 | Domestic | 18°14′44.9''S  63°11′26.8″W  513m | 2012 | Rabbit | BolNat-A  BolNat-N | T.inf-CH2A |
| 51 | Bolivia | Santa Cruz/ Cordillera | Boyuibe/ Pueblo Nuevo | Peri-domestic | 20°24′54.7''S  63°17′13.2″W  812m | 2012 | - | - | - |
| 52 | Bolivia | Santa Cruz/ Cordillera | Boyuibe/ Kuruyuki | Peri-domestic | 20°24′49.7''S  63°24′4.5″W  931m | 2012 | - | - | - |
| 53 | Bolivia | Santa Cruz/ Cordillera | Charagua/  Cuarienda | Domestic | 19°10′48.3''S  62°31′35.5″W  377m | 2012 | - | - | - |
| 54 | Bolivia | Santa Cruz/ Cordillera | Boyuibe/ Pozo del Monte | Peri-domestic | 20°24′58.2''S  63°24′3.9″W  928m | 2012 | Guinea pig | BolNat-A  BolNat-N | T.inf-CH2A |
| 55 | Bolivia | Santa Cruz/ Cordillera | Boyuibe/ Benemerito | Domestic | 20°27′13.6''S  63°16′39.5″W  817m | 2012 | - | - | - |
| 56 | Bolivia | Cochabamba/ Quillacollo | Sipe Sipe/ Urinzaya | Peri-domestic | 17°27′1.4''S  66°21′41.2″W  2600m | 2012 | Rabbit | BolNat-A  BolNat-N | T.inf-CH1A |
| 57 | Bolivia | Cochabamba/ Quillacollo | Sipe Sipe/ Molle-Molle | Peri-domestic | 17°27′39.0''S  66°17′12.6″W  2580m | 2012 | - | - | - |

Supplemental Table S2. Development times of the different developmental stages of *T. infestans* and numbers of triatomines used in feeding experiments

| Developmental stage | Development time of nymphs/female eggs after blood meal (days)* | No. of triatomines/  strain (incl. starved) | Total no. triatomines of all 14 strains |
| --- | --- | --- | --- |
| 1^st^ nymphal stage | 12 | 18 | 252 |
| 2^nd^ nymphal stage | 12 | 18 | 252 |
| 3^rd^ nymphal stage | 15 | 22 | 308 |
| 4^th^ nymphal stage | 17 | 24 | 336 |
| 5^th^ nymphal stage | 28 | 34 | 476 |
| Female | 12 | 18 | 252 |
| Total | - | 134 | 1876 |

* Development times of *T. infestans* and oviposition after a blood meal are in accordance with Schaub et al. (2008), Schofield (1994) and Lucius and Loos-Frank (1997).
