# Supplementary material for: An Updated Insight into the Sialotranscriptome of Triatoma infestans: Developmental Stage and Geographic Variations
Source: PLoS Negl Trop Dis. 2014 Dec 4;8(12):e3372. doi: 10.1371/journal.pntd.0003372 (PMC4256203; doi:10.1371/journal.pntd.0003372)
Supplement: Table S2 — Development times of the different developmental stages of T. infestans and numbers of triatomines used in feeding experiments. (DOCX) [file pntd.0003372.s003.docx]

Supplemental Table S2. Development times of the different developmental stages of *T. infestans* and numbers of triatomines used in feeding experiments

| Developmental stage | Development time of nymphs/female eggs after blood meal (days)* | No. of triatomines/  strain (incl. starved) | Total no. triatomines of all 14 strains |
| --- | --- | --- | --- |
| 1^st^ nymphal stage | 12 | 18 | 252 |
| 2^nd^ nymphal stage | 12 | 18 | 252 |
| 3^rd^ nymphal stage | 15 | 22 | 308 |
| 4^th^ nymphal stage | 17 | 24 | 336 |
| 5^th^ nymphal stage | 28 | 34 | 476 |
| Female | 12 | 18 | 252 |
| Total | - | 134 | 1876 |

* Development times of *T. infestans* and oviposition after a blood meal are in accordance with Schaub et al. (2008), Schofield (1994) and Lucius and Loos-Frank (1997).
