# Supplementary material for: An Updated Insight into the Sialotranscriptome of Triatoma infestans: Developmental Stage and Geographic Variations
Source: PLoS Negl Trop Dis. 2014 Dec 4;8(12):e3372. doi: 10.1371/journal.pntd.0003372 (PMC4256203; doi:10.1371/journal.pntd.0003372)
Supplement: File S1 — Excel spreadsheet reporting 11,188 coding sequences (CDS) hyperlinked to various databases, and mapped reads and polymorphism from 10 different libraries. This file should be used for sorting on appropriate fields to identify over/under expressed CDS. Alternatively, Open Office can be used and can be freely downloaded from http://www.openoffice.org/download/. For the hyperlinks to work, make sure local settings allow hyperlinks from exon.niaid.nih.gov to be accessed. http://exon.niaid.nih.gov/transcriptome/T_infestans/T_infestans-S1.xlsx (DOCX) [file pntd.0003372.s004.docx]

**Supplemental file S1:** Excel spreadsheet reporting 11,188 coding sequences (CDS) hyperlinked to various databases, and mapped reads and polymorphism from 10 different libraries. This file should be used for sorting on appropriate fields to identify over/under expressed CDS.

<http://exon.niaid.nih.gov/transcriptome/T_infestans/T_infestans-S1.xlsx>
