# Supplementary material for: An Updated Insight into the Sialotranscriptome of Triatoma infestans: Developmental Stage and Geographic Variations
Source: PLoS Negl Trop Dis. 2014 Dec 4;8(12):e3372. doi: 10.1371/journal.pntd.0003372 (PMC4256203; doi:10.1371/journal.pntd.0003372)
Supplement: File S2 — Same as S1, but with CDS functionally sorted. http://exon.niaid.nih.gov/transcriptome/T_infestans/T_infestans-S2.xlsx (DOCX) [file pntd.0003372.s005.docx]

**Supplemental file S2:** Same as S1, but with CDS functionally sorted.

<http://exon.niaid.nih.gov/transcriptome/T_infestans/T_infestans-S2.xlsx>
